# Correlation between Serum Levels of 3,3ʹ,5ʹ-Triiodothyronine and Thyroid Hormones Measured by Liquid Chromatography-Tandem Mass Spectrometry and Immunoassay

**DOI:** 10.1371/journal.pone.0138864

**Published:** 2015-10-01

**Authors:** Hiroyuki Sakai, Hidenori Nagao, Mamoru Sakurai, Takako Okumura, Yoshiyuki Nagai, Junpei Shikuma, Rokuro Ito, Tetsuya Imazu, Takashi Miwa, Masato Odawara

**Affiliations:** 1 Department of Diabetes, Endocrinology and Metabolism, Tokyo Medical University, Tokyo, Japan; 2 Pharmacokinetics Research Department of ASKA Pharmaceutical Co., Ltd., Kawasaki, Kanagawa, Japan; University Claude Bernard Lyon 1, FRANCE

## Abstract

**Objective:**

For measuring serum 3,3**′**,5**′**-triiodothyronine (rT3) levels, radioimmunoassay (RIA) has traditionally been used owing to the lack of other reliable methods; however, it has recently become difficult to perform. Meanwhile, liquid chromatography-tandem mass spectrometry (LC-MS/MS) has recently been attracting attention as a novel alternative method in clinical chemistry. To the best of our knowledge, there are no studies to date comparing results of the quantification of human serum rT3 between LC-MS/MS and RIA. We therefore examined the feasibility of LC-MS/MS as a novel alternative method for measuring serum rT3, thyroxine (T4), and 3,5,3′-triiodothyronine (T3) levels.

**Methods:**

Assay validation was performed by LC-MS/MS using quality control samples of rT3, T4, and T3 at 4 various concentrations which were prepared from reference compounds. Serum samples of 50 outpatients in our department were quantified both by LC-MS/MS and conventional immunoassay for rT3, T4, and T3. Correlation coefficients between the 2 measurement methods were statistically analyzed respectively.

**Results:**

Matrix effects were not observed with our method. Intra-day and inter-day precisions were less than 10.8% and 9.6% for each analyte at each quality control level, respectively. Intra-day and inter-day accuracies were between 96.2% and 110%, and between 98.3% and 108.6%, respectively. The lower limit of quantification was 0.05 ng/mL. Strong correlations were observed between the 2 measurement methods (correlation coefficient, T4: 0.976, *p* < 0.001; T3: 0.912, *p* < 0.001; rT3: 0.928, *p* < 0.001).

**Conclusions:**

Our LC-MS/MS system requires no manual cleanup operation, and the process after application of a sample is fully automated; furthermore, it was found to be highly sensitive, and superior in both precision and accuracy. The correlation between the 2 methods over a wide range of concentrations was strong. LC-MS/MS is therefore expected to become a useful tool for clinical diagnosis and research.

## Introduction

Liquid chromatography-tandem mass spectrometry (LC-MS/MS), which has increasingly been used in recent years, is a representative method of mass spectrometry. For the measurement of hormone levels, LC-MS/MS is superior to the commonly used immunoassay, in terms of sensitivity and specificity [1]. LC-MS/MS is further anticipated to have great potential because of its efficacy in measuring small sample volumes and its high specificity. In the field of endocrine hormones, LC-MS/MS is clinically used to measure steroid hormones and vitamin D, and recently, it has also been used to measure the levels of thyroid hormones [1].

When thyroid dysfunction is suspected, the levels of serum thyrotropin, free thyroxine (FT4), and free 3,5,3′-triiodothyronine (FT3) are routinely measured. If additional information is required, such as for differentiating between hypothyroidism and non-thyroidal illness (NTI), 3,3′,5′-triiodothyronine (rT3) measurements are useful [2].

We previously measured rat serum rT3 levels using On-line Solid-Phase Extraction (SPE) LC-MS/MS [3]. Although the measurement of rT3 levels using MS/MS has been reported [4–7], these studies only obtained the results by detection of reference compounds, and hence were not successful in measuring them quantitatively. It is more favorable to measure serum rT3 simultaneously with T4 and T3; however, the levels of serum rT3 are extremely low, and hence require a highly sensitive measurement method. To the best of our knowledge, there have been no studies to date that examined a substantial number of human serum samples and compared LC-MS/MS with the most commonly used immunoassay for rT3.

With regard to serum T4 and T3 levels, there have been several studies comparing measurements obtained by mass spectrometry and those by immunoassay [8–13]. In 4 of these studies [8–11], the same measurement system was used, in which the lower limit of quantification (LLOQ) was 0.15 ng/mL, and serum T4 and T3 were detected with high sensitivity. However, in the other studies [12,13], T3 could not be measured because of the low sensitivity of the assay used. These above studies had limitations, such as the lack of information on characteristics of the subjects [8, 12, 13], as well as the lack of measurements of low concentration samples [8–10, 12, 13]. Furthermore, these studies failed to detect a sufficient correlation between the measurements obtained by MS/MS and those by immunoassay for T3. To demonstrate the feasibility of using LC-MS/MS for disease diagnosis, a wide range of data, including that for both high and low concentrations, should be obtained simultaneously for T4 and T3.

Currently, several types of non-radioimmunoassay (non-RIA) methods are widely used for measuring serum T4 and T3 levels. However, for rT3, RIA is the only reliable method of measurement. Recently, there has been a shift from using RIA to non-RIA methods for hormone measurements. The use of RIA will continue to decline in the future, considering the issue of radioactive material management. Under these circumstances in which measurement of rT3 using RIA is becoming more difficult, it is important to compare and evaluate data obtained from LC-MS/MS using human serum samples with those obtained from RIA, to establish the validity of and to encourage the acceptance of LC-MS/MS as a next-generation assay.

In this study, we modified the LC-MS/MS measurement system that we previously established [3], to enable its application to human serum samples. This measurement system also aimed to improve the LLOQ for rT3 quantification. With the LC-MS/MS system, measurement of serum levels of FT4 and FT3 is laborious and requires additional procedures. With our present LC-MS/MS system, we are able to measure T4 and T3 but not FT4 and FT3. We therefore performed experiments to measure T4 and T3 simultaneously in addition to rT3 using various patient serum samples with an expected wide range of concentrations of these hormones, to examine the correlation between the concentrations obtained by our novel LC-MS/MS system and those obtained by conventional immunoassay.

## Materials and Methods

### Subjects

To select patients with a wide range of concentrations of serum T4, T3, and rT3, we utilized serum FT4 levels, which usually correlate with T4, T3, and rT3 levels. Fifty outpatients (9 men and 41 women), whose FT4 levels were previously measured in our department by the electrochemiluminescenceimmunoassay (ECLIA) method during routine examination, and who had a range of low to high FT4 values were selected by a clinical technologist who was unaware of their backgrounds. Pregnant women were excluded from the study. The patients were 51.7 ± 16.3 years of age (men: 55.4 ± 18.7 years; women: 50.7 ± 16.0 years), and included 35 patients with Graves’ disease, 20 patients who were receiving antithyroid drugs, 8 patients who were receiving potassium iodide, and 13 patients who l-thyroxine replacement therapy (including some overlap).

This study was approved by the ethics committee of Tokyo Medical University (reference number 2699). All patients provided written informed consent before participation in the study.

### Chemicals and reagents

**Fig 1:**
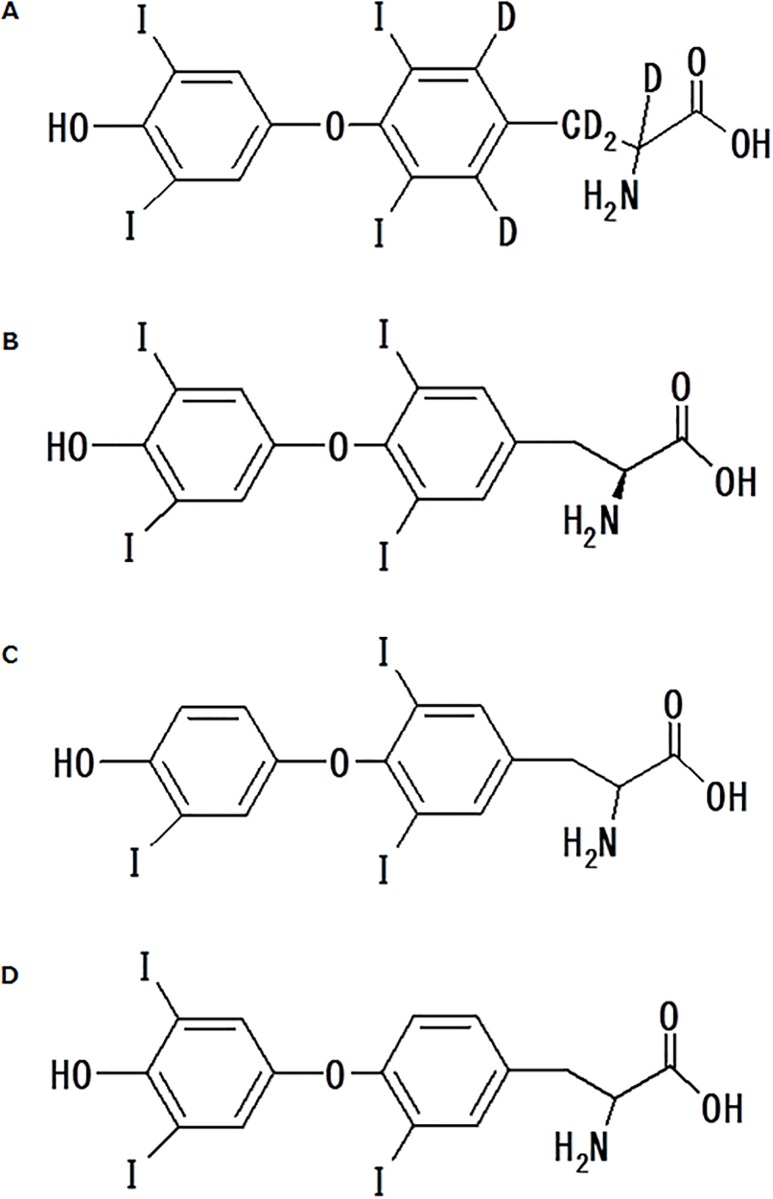
Chemical structures of [^2^H_5_]T4 (A), T4 (B), T3 (C), and rT3 (D).

L-Thyroxine-[L-tyrosine-^2^H_5_] (Fig 1A) was purchased from IsoSciences, LLC (King of Prussia, PA). T4, T3_,_ and rT3 (Fig 1B, 1C and 1D, respectively) were purchased from Sigma-Aldrich Co. (Piscataway, NJ). Thyroid hormone-free human serum was purchased from Golden West Biologicals (DDC Mass Spect Gold^®^, Temecula, CA). All other commercially available chemicals and reagents were of the highest analytical grade.

### Solutions and standards

Stock solutions of T4, T3, and rT3 were each prepared separately to a concentration of 10 μg/mL. Stock solutions of the internal standard (IS, [^2^H_5_]T4) was prepared at 100 μg/mL. Methanol / 25% ammonia solution (98:2) was used as a solvent. Analyte stock solutions were diluted with DDC Mass Spec Gold^®^ to obtain the calibration standards (0.05, 0.1, 0.5, 1, 5, 10, 50, and 100 ng/mL). The IS stock solution was diluted with acetonitrile to obtain the spiking solution (2 ng/mL). All stock solutions were stored at 4°C.

### Sample preparation

A 20-μL aliquot of patient serum (or patient serum diluted with DDC Mass Spec Gold^®^) was mixed with 60 μL of IS spiking solution. After vortex mixing, the solution was centrifuged (13,000 rpm, 5 min, 10°C). Then, 40 μL of 0.1 vol% formic acid was added to the mixture and vortex mixed. After centrifugation (13,000 rpm, 5 min, 10°C), 70 μL of the supernatant was injected into the LC-MS/MS system. Eight calibration standard samples ranging from 0.05 to 100 ng/mL were prepared from DDC Mass Spec Gold^®^. Similarly, quality control samples of T4, T3, and rT3 were prepared at concentrations of 0.05, 0.1, 2.5, and 80 ng/mL. These concentrations were chosen to demonstrate the precision and accuracy of the method at the LLOQ as well as at low, medium, and high concentrations on the calibration curve.

### On-line SPE LC-MS/MS

The high-performance liquid chromatography (HPLC) system (Shimadzu, Kyoto, Japan) consisted of an SCL-10Avp system controller, 3 LC-20AD pumps connected to an FCV-11AL reservoir selection valve, an SIL-HTc autosampler, and a CTO-20A column oven equipped with an FCV-12AH 6-port switching valve for on-line extraction. The SPE column was a Shim pack MAYI-ODS, 2.0 mm I.D. × 10 mm, 50 m (Shimadzu) maintained at 45°C. The analytical column was a Synergi Polar-RP 80A, 2.0 mm I.D. × 50 mm, 4 m (Phenomenex, Utrecht, Netherlands) maintained at 45°C. The mobile phases were 1.0 vol% formic acid (A-1), 0.05 vol% acetic acid (A-2), methanol (B), and 0.05 vol% acetic acid/methanol (95:5, v/v). The LC conditions are listed in Table 1. An API5000 triple quadrupole mass spectrometer (AB SCIEX, Foster City, CA) equipped with a TurboIonSpray source was operated in the positive ion multiple reaction monitoring (MRM) mode to perform the analysis. The transitions to monitor were selected for each compound as follows: *m/z* 777.70 > 604.90 for T4, *m/z* 651.77 > 605.90 for T3, *m/z* 651.77 > 507.73 for rT3, and *m/z* 782.75 > 735.90 for [^2^H_5_]T4.

**Table 1:** Liquid chromatography conditions used for the On-line Solid-Phase Extraction liquid-chromatography tandem mass spectrometry analysis.

Time (min)	Flow rate (mL/min)	FCV-12AH valve position	Mobile phase		
			Pump A		Pump B
			A-1 (%)	A-2 (%)	B (%)
0.00	3.50	A	95	0	5
0.99	4.00	A	95	0	5
1.00	4.00	A	0	95	5
1.80	0.35	A	0	95	5
2.00	0.35	B	0	95	5
2.50	0.35	B	0	95	5
8.00	0.35	B	0	20	80
9.60	0.35	B	0	20	80
9.61	0.35	B	0	1	99
9.63	0.80	B	0	1	99
11.00	0.80	A	0	1	99
11.02	5.00	A	0	1	99
11.03	5.00	A	1	0	99
12.01	5.00	A	95	0	5
12.04	4.00	A	95	0	5
12.54	4.00	A	95	0	5

Valve position A: sample extraction or washing on the Solid-Phase Extraction column

Valve position B: back-flush onto the analytical column and chromatographic separation

Mobile phase A-1: 1.0 vol% formic acid

Mobile phase A-2: 0.05 vol% acetic acid

Mobile phase B: methanol

Pump C delivered 0.05 vol% acetic acid/methanol (95:5, v/v) at 0.35 mL/min to equilibrate the analytical column

### Method validation

#### Matrix effects

Matrix effects were evaluated using 6 individual plasma samples. Plasma samples were spiked with T4, T3, and rT3, each at a concentration of 10 ng/mL. Accuracy was calculated by comparison of the nominal and observed values of concentrations of T4, T3, and rT3 in each spiked sample.

#### Linearity

Calibration curves were prepared by adding a known amount of T4, T3, and rT3 as well as IS to 0.02 mL of DDC Mass Spec Gold^®^. Calibration curves were constructed by plotting the peak area ratio of each T4, T3, and rT3 standard concentrations and of IS in the Y-axis and X-axis concentration ranges. Linearity was assessed using the coefficient of correlation (*r* ^*2*^) values of the linearity plots.

Concentrations of the unknown samples were calculated from the weighted (1/x^2^) linear regression analysis of the standard curve.

#### Precision and accuracy

Inter-day and intra-day precisions were analyzed using the quality control samples (0.05, 0.1, 2.5, and 80 ng/mL) by the On-line SPE LC-MS/MS method. The intra-day accuracy was evaluated by analyzing 5 samples at each concentration and the inter-day accuracy by comparing samples processed on 3 different days. Precision was determined utilizing these same samples.

### Measurements of T4, T3, and rT3 concentrations in patient serum samples

T4, T3, and rT3 concentrations in the stored serum samples were measured simultaneously using On-line SPE LC-MS/MS. For the immunoassay, T4 and T3 concentrations were measured by ECLIA, and rT3 concentration was measured by RIA, as described below.

#### ECLIA

Elecsys (Roche Diagnostics GmbH, Mannheim, Germany) was used for ECLIA. The method for measuring T4 concentration had a sensitivity of 0.04 μg/dL and a precision of less than 4% at all concentrations tested, and was calibrated for the range of 0.42 to 24.9 μg/dL. The method for measuring T3 concentrations had a sensitivity of 0.03 ng/mL and a precision of less than 6% at all concentrations tested, and was calibrated for the range of 0.20 to 6.51 ng/mL.

#### RIA

Serum rT3 concentrations were determined using RIA kit (RIAZEN Reverse T3^®^, ZenTech, Angleur, Belgium) in accordance with the manufacturer’s instructions. All measurements were performed in duplicate. The sensitivity of the determinations was 0.009 ng/mL. The intra-assay and inter-assay coefficients of variation were both less than 9% using the samples (0.12–0.72 ng/mL).

### Statistical analysis

Statistical associations between the results obtained by LC-MS/MS and those obtained by immunoassay were evaluated using Spearman correlation coefficients, using SPSS version 17 (SPSS Inc., Chicago, IL).

## Results

### Assay validation of On-line SPE LC-MS/MS

#### LC-MS/MS characteristics

Typical chromatograms of T4, T3, rT3, and [^2^H_5_]T4 in a patient serum sample are shown in S1 Fig. The peaks corresponding to T4, T3, rT3, and [^2^H_5_]T4 with acceptable shapes were clearly observed at 8.7, 8.3, 8.5, and 8.7 min, respectively.

#### Matrix effects

The matrix effects were evaluated by the accuracy and precision of measurement of the nominal and observed values of concentrations of analytes, and all of the values were within 100 ± 15% and 15%, respectively. A detailed summary of the accuracy and precision data is shown in S1 Table. The analysis of 6 individual plasma samples did not demonstrate any significant interference from matrix components.

#### Linearity

Correlation coefficients (*r* ^*2*^) between the concentrations of the T4, T3, and rT3 standards obtained by On-line SPE LC-MS/MS and the ratios of their peak area were 0.996, 0.999, and 0.999, respectively, which demonstrated good linear relationships. The calibration curves generated the following regression equations: y = 0.203x + 0.00322 for T4, y = 1.36x + 0.000547 for T3, and y = 0.664x + 0.00607 for rT3. The lowest concentration on these curves was defined as the LLOQ for all the analytes, and was established to be 0.05 ng/mL.

#### Accuracy and precision

For T4_,_ T3, and rT3, intra-day and inter-day measurement precision and accuracy were evaluated by analysis of the 0.05, 0.1, 2.5, and 80 ng/mL quality control samples. The results are summarized in Tables 2 and 3. Overall, intra-day and inter-day precisions were less than 10.8% and 9.6% for each analyte at each quality control level, respectively. Accuracies were between 96.2% and 110%, and between 98.3% and 108.6%, for intra-day and inter-day measurements, respectively. Therefore, the LLOQ of each analyte was established as 0.05 ng/mL. These results indicated that On-line SPE LC-MS/MS is a reliable technique for the precise and accurate measurement of serum thyroid hormones. This LC-MS/MS system enables the preparation of samples with an automated cleanup function. Moreover, this system enables fully automated quantification of a sample after its application to the device, by the on-line SPE function.

**Table 2:** Precision and Accuracy of intra-day assay.

Concentration (ng/mL)	Accuracy (%)					
	T4		T3		rT3	
0.05	104.0±8.0	(7.7)	110.0±4.0	(3.6)	110.0±4.0	(3.6)
0.1	105.0±6.0	(5.7)	104.0±7.0	(6.7)	104.0±7.0	(6.7)
2.5	108.1±3.1	(2.9)	100.3±5.5	(5.5)	100.3±5.5	(5.5)
80	102.5±1.3	(1.3)	96.2±6.6	(6.9)	96.2±6.6	(6.9)

Data are expressed as the mean ± S.D. (n = 5).

Values in parentheses are coefficients of variance (%).

**Table 3:** Precision and Accuracy of inter-day assay.

Concentration (ng/mL)	Accuracy (%)					
	T4		T3		rT3	
0.05	104.0±10.0	(9.6)	108.0±6.0	(5.6)	106.0±6.0	(5.7)
0.1	104.0±6.0	(5.8)	107.0±6.0	(5.6)	106.0±8.0	(7.5)
2.5	108.6±3.1	(2.9)	105.6±5.3	(5.0)	107.4±5.3	(4.9)
80	102.1±2.0	(1.9)	98.3±4.5	(4.5)	102.6±5.0	(4.9)

Data are expressed as the mean ± S.D. (n = 15).

Values in parentheses are coefficients of variance (%).

### Measurement of patient serum samples

**Fig 2:**
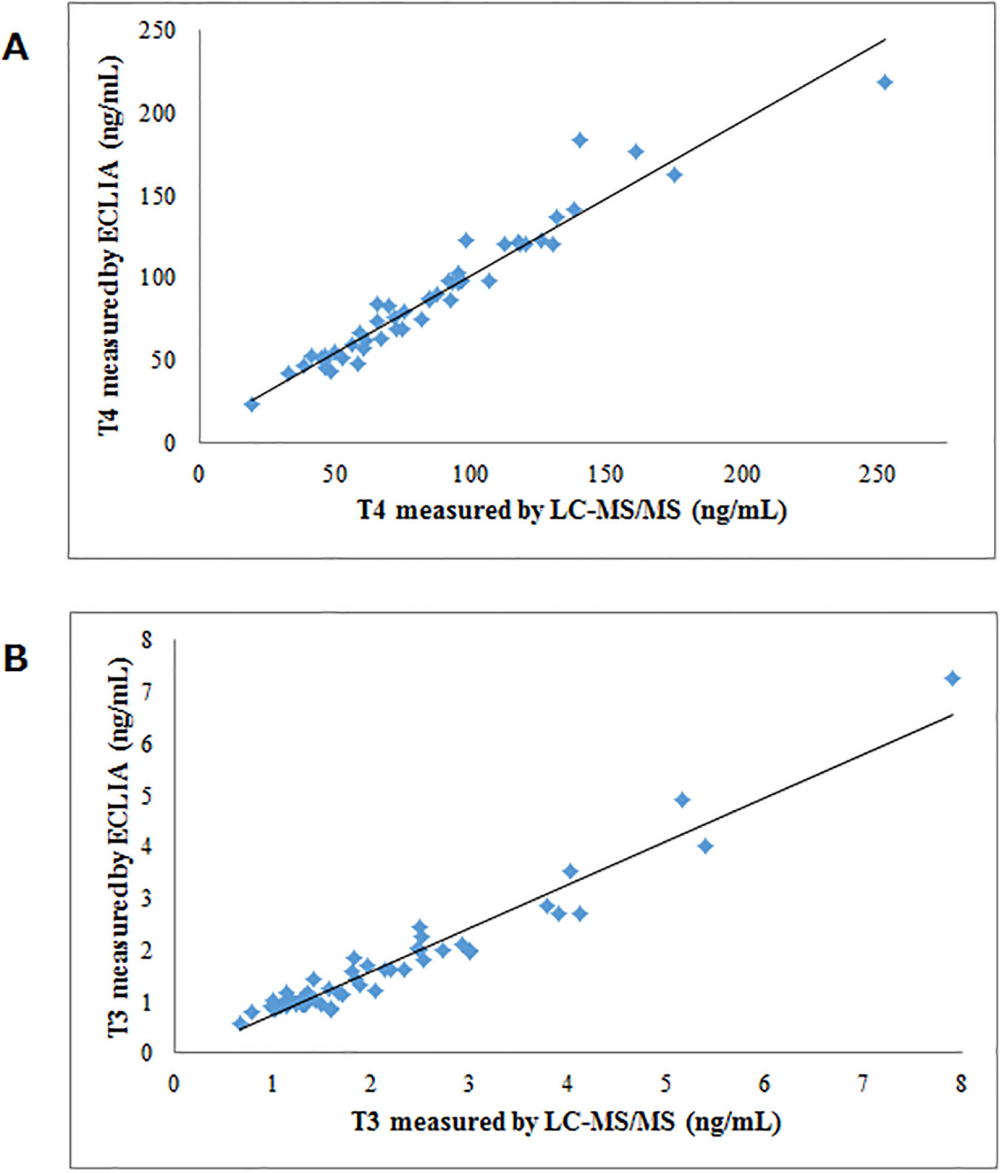
Comparison of the LC/MS/MS method with the ECLIA method for the measurement of serum T4 (A) and T3 (B). (A) y = 0.94x + 8.24, *r* = 0.976, *p* < 0.001; (B) y = 0.84x + 0.12, *r* = 0.912, *p* < 0.001.

**Fig 3:**
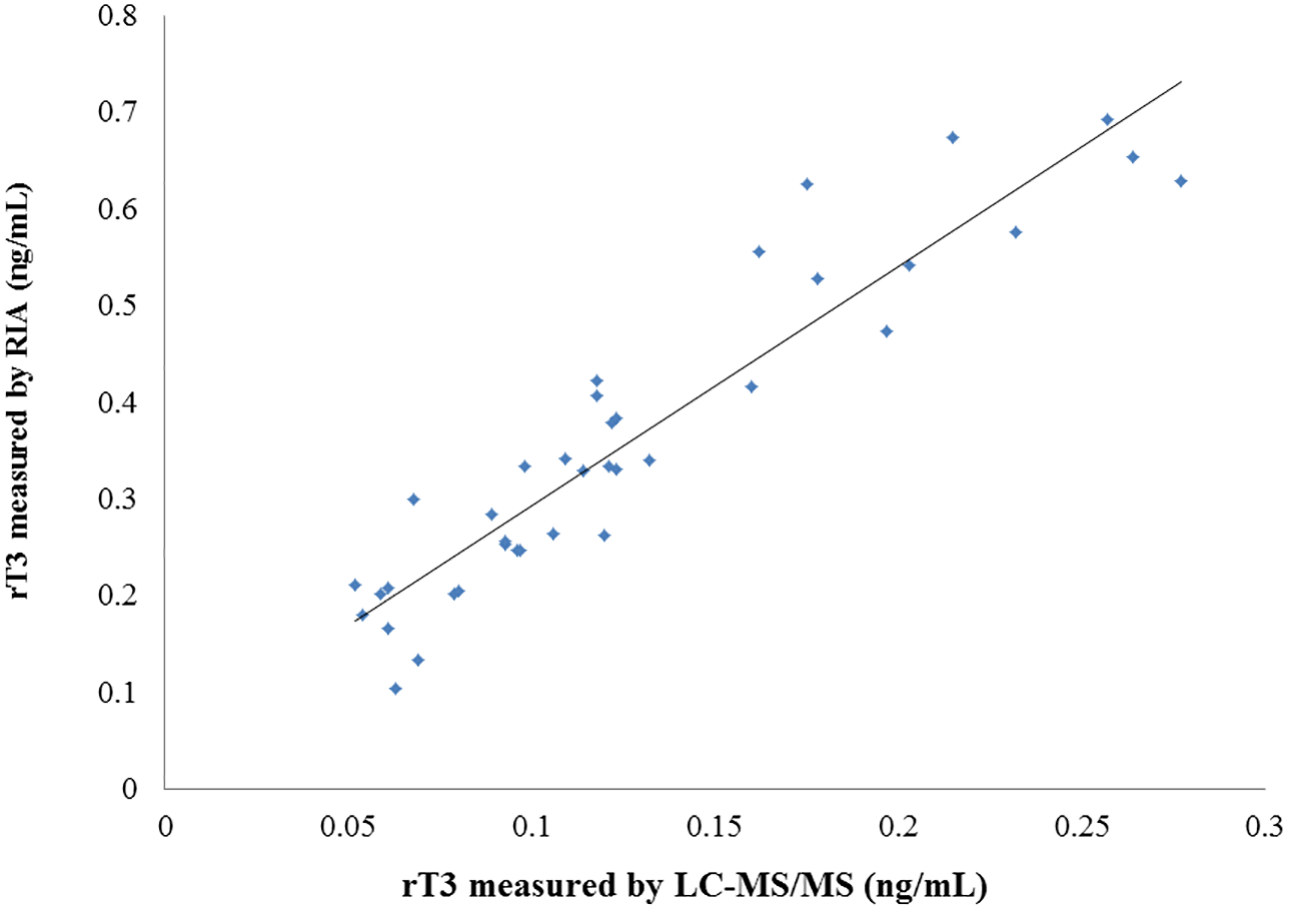
Comparison of the LC/MS/MS method with the RIA method for the measurement of serum rT3. y = 2.48x + 0.0456, *r* = 0.928, *p* < 0.001.

For serum T4 and T3 levels determined by LC-MS/MS and ECLIA, very strong correlations between the 2 measurement methods were obtained (*r* = 0.976, *p* < 0.001; *r* = 0.912, *p* < 0.001, respectively) (Fig 2A and 2B). Regarding the measurements of rT3 concentrations, 4 samples were found to be lower than 0.05 ng/mL (LLOQ) by LC-MS/MS, and 8 samples were higher than 0.72 ng/mL (maximum concentration of precision) by RIA. We therefore excluded those 12 samples, and statistically analyzed the remaining data (n = 38). A strong correlation was also observed between the measurements obtained by LC-MS/MS and by RIA (*r* = 0.928, *p* < 0.001) (Fig 3).

## Discussion

In this study, we compared the results of LC-MS/MS used as a next-generation measurement method for human serum thyroid hormones to those of conventional immunoassay and obtained a strong correlation between the 2 measurement methods.

Serum rT3 levels are useful data for the diagnosis of NTI, differentiation between central hypothyroidism and NTI, and pathological diagnosis of neonatal hyperthyrotropinemia [2, 13]. Moreover, there are many clinical studies showing that serum rT3 levels and the T3/rT3 ratio are associated with the prognosis of critical illnesses [2, 14, 15]. In such clinical studies, it is necessary to measure serum levels of rT3, as well as T4 and T3, to gain more information about the diseases. Even in recent studies measuring rT3 [16, 17], measurements were made by RIA, which is the most reliable but very old technique [18]. However, the number of studies on rT3 has substantially decreased in recent years. This may be owing to the RIA kit for rT3 being difficult to use.

Currently, several types of immunoassay are used to measure serum levels of T4 and T3. There are substantial differences between measurement methods based on different principles and reagents, and hence the interpretation of the results requires caution. For measuring steroid hormones [19] and vitamin D (particularly 25-hydroxyvitamin D), the specificity of the currently used immunoassay is markedly low owing to cross-reactivity. Moreover, its level of sensitivity is insufficient for measuring testosterone and estradiol [1]. These limitations apply to immunoassays in general. Furthermore, autoantibodies [20, 21] and heterophilic antibodies (e.g., human anti-mouse antibody) contained in the samples may interfere with immunoassays [22–24]. Owing to the above reasons, MS, particularly LC-MS/MS, is increasingly being used in the field of clinical chemistry. Furthermore, the analysis targets are shifting from conventional drugs and their metabolites to hormones and peptides [1, 22].

MS used for measuring T3 and T4 first started as gas chromatography, followed by isotope dilution mass spectrometry with selected ion monitoring. However, these methods required laborious procedures, such as cleanup and derivatization. Subsequently, substantial progress was achieved in that the LC and MS instruments themselves became high performance. In addition, the specificity of the instruments was improved by addition of the MRM mode [1, 25, 26].

Generally, LC-MS/MS is superior in specificity and sensitivity compared to the immunoassay [1]. However, a substantial amount of skill is required to set up an LC-MS/MS measurement system by combining the optimal conditions for each measurement item. In general, the initial cost is high, and the operation and maintenance of the device require advanced knowledge. There are also drawbacks, such as the necessity to pay attention to ion suppression during analysis. Furthermore, depending on the measurement system, laborious sample preparation is required [1].

On the other hand, our measurement system that we used in this study requires no laborious manual cleanup steps. Moreover, the process after application of a sample to the device is fully automated so that the device can be left unattended. Although matrix effects from various components in biological samples interfered the accuracy of quantitative analytical method, the matrix effects were not observed in our measurement system. Favorable results were obtained with this system in terms of both precision and accuracy for measuring T4, T3, and rT3 levels. Furthermore, compared to the measurement system reported previously [3] and other MS systems for measuring T4 and T3 [8–13], the system used in this study had a further improved LLOQ and allowed sufficient quantification levels of rT3 that are lower than the levels of T3.

Regarding the studies examining the correlation between MS and immunoassay for measuring serum T3 and T4 levels [8–13], a very strong correlation between the methods was obtained for T4 in 2 studies (8, 12; *r* = 0.931 and *r* ^*2*^ = 0.982, respectively). However, 1 of the 2 studies [8] used samples with T4 levels close to the normal range, and the other study did not include any low concentration samples and had incomparable factors such as the methods [12]. Other studies used particular patients according to their specific study objectives, such as post-thyroidectomized patients [11], neonates [13], and pregnant women [9, 10]. Furthermore, to our knowledge, there has been only 1 study demonstrating a strong correlation for T3 [27]. Although the measurement systems used in some of the studies [8–11] had an LLOQ of 0.15 ng/mL, which indicates the ability to measure T3, the correlation was poor, unlike in our study. Possible reasons for this include the particular patients analyzed, differences in measurement methods of the immunoassay (ECLIA used in our study and chemiluminescence immunoassay used in the other studies), and differences in the LC-MS/MS measurement systems themselves. A comparative study of MS showed that if the control method is accurate, results of the analysis will have a correlation coefficient closer to 1. However, because immunoassay (the most commonly used but less accurate method) was used as the control method, we consider that methods with a correlation coefficient above a certain level (approximately 0.9 − 0.95) may be useful in clinical practice as a novel alternative assay. In our present study, using a highly sensitive LC-MS/MS system, we were able to quantify both T4 and T3 levels in samples with a wide range of concentrations, and very strong correlations were obtained between our method and conventional immunoassay (*r* = 0.976 and 0.912, respectively). The most recent study [27] published by the group that published the previous studies examining the correlation between MS and immunoassay for measuring serum T3 and T4 levels [8–11] presented an improved measurement method, in which very strong correlations were obtained between their new method and immunoassay for both T4 and T3 (*r* = 0.91 and 0.95, respectively). However, they reported that measurement discrepancies were still observed at the low and high concentrations measured [27]. In contrast, such discrepancies were not observed in our present study.

Previous reports on attempts to measure rT3 by MS/MS only demonstrated the detection of reference compounds [4, 7]. Quantification was unsuccessful in a study using bovine serum [7]. Likewise, in other reports on the measurement of reference compounds [5, 6], only the T3/rT3 ratio was determined. To the best of our knowledge, there are no studies to date comparing results of the quantification of human serum rT3 between MS/MS and RIA. This may be attributable to the fact that quantification of rT3 requires a highly sensitive measurement system. In general, a novel measurement method should be compared to the most conventional method. The quantification of rT3 by RIA is based on the previous study [28]. In this study, a dose-response curve was shown (y-axis: [^125^I]rT3 bound, x-axis: rT3 concentration). This curve flattened down in high rT3 concentration range (greater than approximately 1 ng/mL), which means that small difference of [^125^I]rT3 bound in the assay would result in great difference in rT3 concentrations. Therefor it is considered to be difficult to accurately quantify rT3 by RIA at high concentrations. LC-MS/MS method has an advantage over RIA in terms of its good linear relationship to extreme high rT3 concentrations. Our present study showed a strong correlation between the results obtained by our new method and conventional RIA (*r* = 0.928) at concentrations lower than 0.72 ng/mL by RIA. However, the slope of the graph was steep. This graph means that the results measured by RIA were higher than those by LC-MS/MS. In the previous study [18], differences of standard compounds resulted in great differences in the results (occasionally as much as 2–3 times). This is considered to be the main reason for the steep slope. Moreover, cross-reactivity of T4 may cause rT3 overestimation [28], and this might also be a reason. On the other hand, less steep slope and a large value at the y-intercept (y = 0.75x + 0.488, data not shown) were obtained for 8 samples with high concentrations by RIA (0.73–1.96 ng/mL). As described above, we excluded the 8 samples that may have been measured inaccurately. Low levels of radioactivity require accurate quantification to enable the accurate evaluation of high rT3 concentrations by RIA, because small overestimation of radioactivity would results in large underestimation of rT3 concentrations.

A limitation of this study may be the inclusion of patients with various thyroid diseases. The primary objective of this study was to examine whether our LC-MS/MS measurement system could achieve practical reliability for measuring samples with both high and low thyroid hormone concentrations. Thus, we included patients with hypothyroidism or thyrotoxicosis, and those receiving antithyroid drugs or l-T4, etc., as study subjects. Although methods applicable for actual laboratory examination should provide consistent performance for the measurement of patients with various diseases, in basic clinical studies, it is preferable to analyze various homogeneous groups, such as patients with a specific disease.

Our new measurement system used in this study achieved an acceptable LLOQ level (0.05 ng/mL), which indicated that the system is sufficient for clinical use for the quantification of rT3. Despite the use of samples with a wide range of concentrations, strong correlations of the measurements with the conventional immunoassay were observed for rT3, T3, and T4, suggesting that LC-MS/MS can be used successfully in actual clinical practice.

## Supporting Information

S1 Fig Typical LC-MS/MS chromatograms of T4 (A), T3 (B), rT3 (C) and [^2^H_5_]T4 (D) in a patient serum sample. The measured concentrations of T4, T3 and rT3 were 65.8, 1.00 and 0.178 ng/mL, respectively.
(TIF)

S1 Table Matrix effects of T4, T3, and rT3. (DOCX)
