# Correction: Correlation between Serum Levels of 3,3ʹ,5ʹ-Triiodothyronine and Thyroid Hormones Measured by Liquid Chromatography-Tandem Mass Spectrometry and Immunoassay

**DOI:** 10.1371/journal.pone.0159169

**Published:** 2016-07-07

**Authors:** Hiroyuki Sakai, Hidenori Nagao, Mamoru Sakurai, Takako Okumura, Yoshiyuki Nagai, Junpei Shikuma, Rokuro Ito, Tetsuya Imazu, Takashi Miwa, Masato Odawara

There are numerical errors in the rT3 columns of Table 2 of the published article. Please see the correct Table 2 here.

**Table 2:** Precision and Accuracy of intra-day assay.

Concentration	Accuracy (%)		
(ng/mL)	T4	T3	rT3
0.05	104.0 ± 8.0 (7.7)	110.0 ± 4.0 (3.6)	108.0 ± 6.0 (5.6)
0.1	105.0 ±6.0 (5.7)	104.0 ± 7.0 (6.7)	102.0 ± 11.0 (10.8)
2.5	108.1 ± 3.1 (2.9)	100.3 ± 5.5 (5.5)	101.8 ± 4.5 (4.4)
80	102.5 ± 1.3 (1.3)	96.2 ± 6.6 (6.9)	98.5 ± 6.4 (6.5)

Data are expressed as the mean ± S.D. (n = 5).

Values in parentheses are coefficients of variance (%).
