# Supplementary figures and images for: Correlation between Serum Levels of 3,3ʹ,5ʹ-Triiodothyronine and Thyroid Hormones Measured by Liquid Chromatography-Tandem Mass Spectrometry and Immunoassay
Source: PLoS One. 2015 Oct 1;10(10):e0138864. doi: 10.1371/journal.pone.0138864 (PMC4591014; doi:10.1371/journal.pone.0138864)

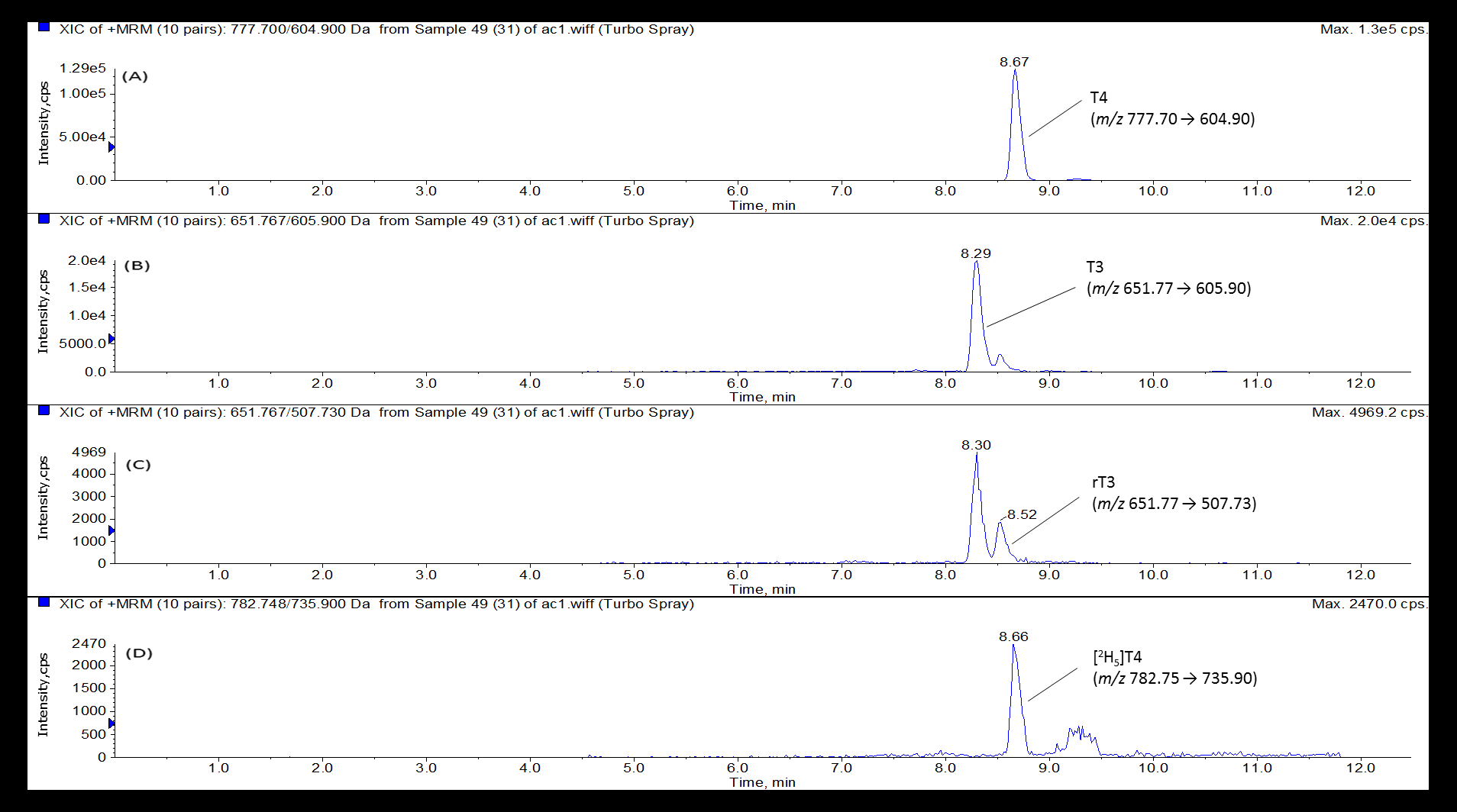

Supplement: S1 Fig — The measured concentrations of T4, T3 and rT3 were 65.8, 1.00 and 0.178 ng/mL, respectively. (TIF) [file pone.0138864.s001.tif]
