# Supplementary material for: Correlation between Serum Levels of 3,3ʹ,5ʹ-Triiodothyronine and Thyroid Hormones Measured by Liquid Chromatography-Tandem Mass Spectrometry and Immunoassay
Source: PLoS One. 2015 Oct 1;10(10):e0138864. doi: 10.1371/journal.pone.0138864 (PMC4591014; doi:10.1371/journal.pone.0138864)
Supplement: S1 Table — (DOCX) [file pone.0138864.s002.docx]

| Analyte | Accuracy (%) | | | | | | Mean | SD | CV (%) |
| --- | --- | --- | --- | --- | --- | --- | --- | --- | --- |
|  | 1 | 2 | 3 | 4 | 5 | 6 |  |  |  |
| T4 | 96.0 | 107.4 | 102.4 | 102.5 | 102.9 | 100.1 | 101.9 | 3.7 | 3.6 |
| T3 | 102.0 | 110.6 | 110.7 | 103.9 | 104.4 | 103.3 | 105.8 | 3.8 | 3.6 |
| rT3 | 105.2 | 110.2 | 103.3 | 109.1 | 115.8 | 106.1 | 108.3 | 4.5 | 4.2 |

**S1 Table. Matrix effects of T4, T3, and rT3**

Matrix effects were evaluated using 6 individual plasma samples. Plasma samples were spiked with T4, T3, and rT3 at a concentration of 10 ng/mL. Accuracy was calculated by comparison of the nominal and observed values of concentrations of T4, T3, and rT3 in each spiked sample. CV; coefficients of variance
